# Supplementary material for: Actual versus Forecast Burden of Primary Hip and Knee Replacement Surgery in Australia: Analysis of Data from the Australian Orthopaedic Association National Joint Replacement Registry
Source: J Clin Med. 2022 Mar 28;11(7):1883. doi: 10.3390/jcm11071883 (PMC8999855; doi:10.3390/jcm11071883)
Supplement: Supplementary file 1 [file jcm-11-01883-s001.zip › jcm-1631997-supplementary.pdf]

Table S1. Comparison of population size* data

	Overall		<40 years		40-69 years		≥70 years	
	**Projected**	**Actual**	**Projected**	**Actual**	**Projected**	**Actual**	**Projected**	**Actual**
**Females**								
2014	9,648,537	9,647,230	4,026,050	4,032,390	4,339,492	4,336,120	1,282,995	1,278,720
2015	9,818,661	9,799,140	4,091,687	4,092,690	4,403,918	4,392,580	1,323,056	1,313,870
2016	9,988,373	9,962,840	4,158,404	4,162,850	4,464,920	4,447,540	1,365,049	1,352,450
2017	10,156,940	10,141,330	4,223,332	4,244,980	4,509,524	4,486,060	1,424,084	1,410,290
2018	10,324,887	10,309,580	4,286,264	4,319,190	4,561,328	4,526,670	1,477,295	1,463,720
2019	10,493,001	10,479,840	4,345,802	4,384,660	4,618,530	4,577,550	1,528,669	1,517,630
**Males**								
2014	9,427,715	9,388,740	4,126,052	4,115,740	4,252,545	4,227,690	1,049,118	1,045,310
2015	9,594,014	9,517,350	4,192,280	4,164,730	4,311,063	4,268,570	1,090,671	1,084,050
2016	9,760,673	9,655,500	4,260,469	4,222,220	4,365,437	4,306,540	1,134,767	1,126,740
2017	9,925,159	9,821,440	4,326,111	4,300,240	4,404,441	4,334,110	1,194,607	1,187,090
2018	10,089,018	9,980,340	4,390,088	4,374,210	4,450,874	4,363,800	1,248,056	1,242,330
2019	10,253,919	10,143,330	4,450,936	4,446,500	4,504,860	4,401,820	1,298,123	1,295,010

* Number of Australians aged 15 years or above
